# Prophylactic Peri-Nephric Drain Placement in Renal Transplant Surgery: A Systematic Review and Meta-Analysis

**DOI:** 10.3389/ti.2024.13030

**Published:** 2024-08-02

**Authors:** Adil S. Lakha, Shahzaib Ahmed, James Hunter, John O’Callaghan

**Affiliations:** ^1^ Oxford University Hospitals National Health Service (NHS) Foundation Trust, Oxford, United Kingdom; ^2^ Nuffield Department of Surgical Sciences, University of Oxford, Oxford, United Kingdom; ^3^ Bristol Royal Infirmary, Bristol, United Kingdom; ^4^ University Hospitals Coventry and Warwickshire, Coventry, United Kingdom; ^5^ Centre for Evidence in Transplantation, University of Oxford, Oxford, United Kingdom

**Keywords:** drain, renal transplant, prophylactic drainage, collection, perinephric drain

## Abstract

Renal transplantation is common worldwide, with >25,000 procedures performed in 2022. Usage of prophylactic perinephric drains is variable in renal transplantation; drains are associated with risks, and there is a lack of consensus regarding benefit of routine drain placement in these patients. This meta-analysis assessed whether prophylactic drainage reduced need for reintervention postoperatively. This systematic review and meta-analysis was carried out using the Preferred Reporting Items in Systematic Reviews and Meta-Analysis, and prospectively registered on PROSPERO. Summary statistics for outcomes of interest underwent meta-analyses to a confidence interval (CI) of 95% and are presented as Forest Plots for Odds Ratio (OR). A systematic literature search in June 2023 revealed 1,540 unique articles across four databases. Of these, four retrospective cohort studies were selected. Meta-analysis of three studies showed no significant reduction in reintervention rate with pre-emptive drain placement, OR = 0.59 (95% CI: 0.16–2.23), *p* = 0.44. Meta-analysis did not show a significant reduction in perinephric collections with prophylactic drain insertion OR = 0.55 (95% CI: 0.13–2.37), *p* = 0.42. Finally, there is not good evidence that drain placement reduces superficial wound complications or improves 12-month graft survival. Further work is needed, including well-designed, prospective studies to assess the risks and benefits of drain placement in these patients.

**Systematic Review Registration**: https://www.crd.york.ac.uk/prospero/display_record.php?ID=CRD42023422685, Identifier PROSPERO CRD42021255795.

## Graphical Abstract

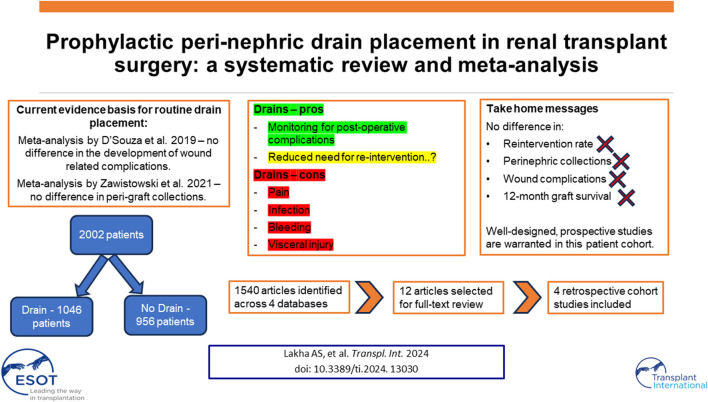

## Introduction

Usage of prophylactic perinephric drains is variable in renal transplantation, and there is a lack of consensus as to the relative benefit of placing an abdominal drain intraoperatively in this patient cohort [1]. Drainage of post-operative fluid collections and prevention of the development of perinephric collections are the main indications for placing such drains in this cohort of immunosuppressed surgical patients [2]. However, there is debate over the necessity of these drains, and whether they may introduce more risks. For example, placement of a drain can result in several complications, including but not limited to post-operative pain, visceral injury, surgical site infection, bleeding or malposition [3, 4]. Prospective studies in general and colorectal surgery have shown a higher surgical site infection risk when drains are inserted intraoperatively [4]. Furthermore, meta-analysis of randomised trials as well as prospective interventional studies suggest drain insertion results in more pain for patients who received intraoperative drain placement [5, 6].

The pathological basis for the development of collections is multifactorial, however immunosuppression, increasing age, obesity, smoking, difficulty of the operation such as bleeding or damage to surrounding structures such as lymphatic tissue in the recipient’s iliac lymph trunk are all thought to contribute to fluid collections post-operatively [7, 8]. Placing a drain during the index transplantation operation therefore is thought to serve as prophylaxis against these relatively common surgical complications. However, these complications are often sub-clinical, may occur after a surgical drain is removed, and not all post-operative collections require drainage. In addition, intraoperative haemostatic techniques may also be utilised to minimise fluid effusion post renal transplantation [9].

This systematic review aims to investigate the impact of prophylactic perinephric drains placed during renal transplantation surgery on immediate and short-term post-operative surgical complication rates. In addition, the broader impact on graft function will be assessed, as well as relevant important outcomes such as deep wound complications, and surgical site infection.

## Methods

This study was carried out following the Preferred Reporting Items in Systematic Reviews and Meta-Analysis (PRISMA) [10]. The protocol was prospectively registered on the PROSPERO system from the University of York (CRD42021255795) on 10th May 2023 [11].

### Literature Search

A literature search was carried out on 1st June 2023, using a combination of Medical Subject Heading (MeSH) terms, free text and keywords to limit the search to renal transplantation operations and drain placement. Complete search strategy is available in Appendix 1. Cochrane protocols, trials and reviews, Transplant Library, Embase, and Medline were all searched on the same date. Each article was assessed using the inclusion criteria outlined below, and any disagreement regarding the eligibility of an article was discussed. Agreement was reached by consensus with a third, and independent, reviewer.

### Inclusion and Exclusion Criteria

There were no language or time-period restrictions. Abstract-only and conference presentation publications were excluded, as were studies assessing paediatric populations and combined transplantation procedures such as simultaneous pancreas-kidney. We included papers which compared outcomes of patients who had a perinephric drain placed intraoperatively during renal transplantation. Patients with drains placed superficial to the musculofascial layer (superficial drain), or patients with drains inserted percutaneously, were excluded.

### Quality Assessment

Methodological quality of included studies was assessed using the Newcastle-Ottawa Score (NOS) tool, a validated scale for assessing the quality of cohort studies [12]. Two independent reviewers performed quality assessment with discrepancies discussed.

### Data Extraction

Data were extracted using a standardised and predesigned data collection form. Data were extracted, where available, on study design characteristics (type of study design, follow-up length), donor kidney type (live or deceased), and outcomes of interest. Post-operative reintervention rate of any kind (either percutaneous image guided drainage, or return to theatre) was the primary outcome for comparison between drain and drain-free patient groups. Additional outcomes such as superficial and deep wound complications, graft survival at 12 months (where available) and delayed graft function were also collected.

### Data Synthesis

Data analyses were performed and figures were extracted from Microsoft Excel and the statistical package RevMan Version 5.8.0, The Cochrane Collaboration, 2020. Heterogeneity was calculated for the meta-analyses using the I^2^ statistic, with the Mantel-Haenszel method and random-effects model utilised due to heterogeneity between the studies.

Summary statistics for outcomes of interest underwent meta-analyses to a confidence interval (CI) of 95% and are presented as Forest Plots for Odds Ratio (OR).

## Results

**FIGURE 1:**
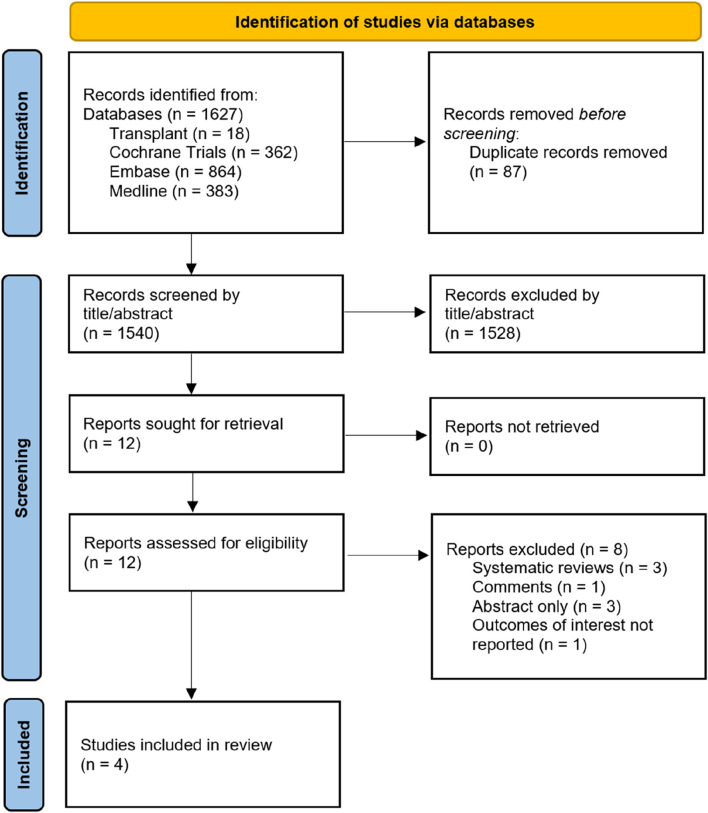
PRISMA flow diagram.

Across all four databases, 1,627 papers were identified, of which 87 were identified as duplicates and discarded. Our search therefore revealed 1,540 unique titles and abstracts across all four databases. Of these, four retrospective cohort studies were selected according to the methodology outlined above, and these are presented in Table 1. Figure 1 outlines a PRISMA flow diagram in selecting articles for inclusion. Across the four studies selected, a total of 2,002 patients’ outcomes data were extracted for analysis. 1,046 had an intraoperative drain placed, 956 did not. Drains were removed when the output recorded less than <50 mL/24 h consistently across three of the studies, and was not reported in the remaining study. Only Farag et al. reported the type of drain used (a Jackson-Pratt suction drain). Furthermore, three out of the four studies reported complete data on type of donor (live vs. deceased), Table 1.

**TABLE 1:** Summary of studies included, and overall recommendations regarding prophylactic drainage.

				Drain insertion donor type		No drain insertion donor type		
Study	Methodology	Drain insertion, n	No drain insertion, n	Live donor (%)	Deceased donor (%)	Live donor (%)	Deceased donor (%)	Overall recommendation
Derweesh et al.	Single centre, retrospective cohort study	81	84	56	44	64	36	Use drain in patients receiving sirolimus
Cimen et al.	Single centre, retrospective cohort study	374	283	38	62	39	61	No benefit with drain insertion
Farag et al.	Single centre, retrospective cohort study	112	388	13	87	42	58	No benefit with drain insertion
Sidebottom et al.	Single centre, retrospective cohort study	479	201	—	—	—	—	No benefit with drain insertion

### Quality Assessment

Methodological quality of included studies was assessed using the Newcastle-Ottawa Score (NOS), and Table 2 shows all included studies and their respective quality assessments. All studies were rated as “good quality” when NOS scores were converted to Agency for Healthcare Research and Quality (AHRQ) descriptors according to the following threshold: three or four stars in the selection domain, and one or two stars in the comparability domain, and two or three stars in the outcome/exposure domain.

**TABLE 2:** Quality assessments using the NOS.

Study	Selcection of cohorts				Comparability	Outcome		
	Representativeness of the exposed cohort	Selection of the non-exposed cohort	Ascertainment of exposure	Demonstration that outcome of interest was not present at start of study	Comparability of cohorts on the basis of the design or analysis	Assessment of outcome	Was follow up long enough for outcomes to occur	Adequacy of follow up of cohorts
Derweesh et al. 2008 [2]	☆	☆	☆	☆	☆	☆	☆	☆
Sidebottom et al. 2014 [13]	☆	☆	☆	☆	☆	☆	—	☆
Cimen et al. 2016 [14]	☆	☆	☆	☆	☆ ☆	☆	—	☆
Farag et al. 2021 [15]	☆	—	☆	☆	☆	☆	☆	☆

### Reintervention Rate

**FIGURE 2:**
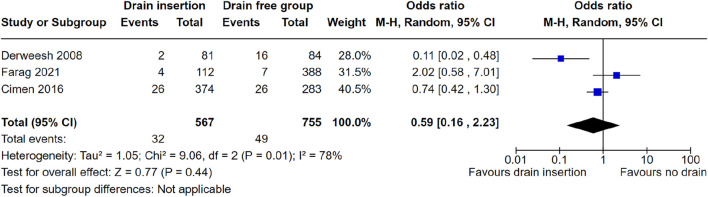
Meta-analysis of reintervention rate.

We performed a meta-analysis to ascertain whether intraoperative perinephric drain placement was associated with a reduced need for either image-guided percutaneous drainage or return to theatre post renal transplantation. Meta-analysis of three studies showed no evidence of a significant reduction in reintervention rate with drain placement, OR = 0.59 (95% CI: 0.16–2.23), *p* = 0.44, Figure 2. The study from Sidebottom et al. did not report reintervention rate post renal transplant, therefore was not included in the meta-analysis.

### Deep Wound Complications

**FIGURE 3:**
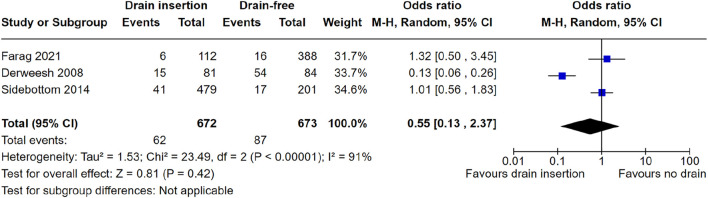
Meta-analysis of deep wound complications.

Three studies reported figures for deep wound complications and were therefore included for meta-analysis. Meta-analysis did not show a significant reduction in perinephric collections with prophylactic drain insertion OR = 0.55 (95% CI: 0.13–2.37) *p* = 0.42, Figure 3. One study could not be included in meta-analysis as they only reported an odds ratio (rather than raw patient-level data) for reduced risk, favouring drain insertion due to lower rates of peri-graft collections OR = 0.62 (95% CI: 0.43–0.88), *p* = 0.01.

### Superficial Wound Complications

Only two studies reported the rates of superficial wound complications with a standardised definition, with superficial complications inclusive of wound evisceration, infection and dehiscence. Derweesh et al. reported no significant difference between the percentage of wound complications in the drain (13.6%) and no drain group (22.6%), *p* = 0.13. Farag et al. reported superficial wound complications (inclusive of subcutaneous seroma or wound dehiscence), with no statistically significant difference in the incidence of wound complications between the drain and drain-free groups (*p* = 0.35).

### Graft Survival at 12 months

Finally, we intended to assess graft survival at 12 months and whether or not there was any difference between drain and drain-free cohorts. Sidebottom et al. reported a 30 days follow up, and Cimen et al. reported 1 month longest follow up data. Farag et al. reported 98.5% and 96.4% graft survival rates in drain-free and drainage groups, respectively (*p* = 0.20). Similarly, Derweesh et al. reported graft survival rates of 83% and 88% in drain-free and drainage groups, respectively (*p* = 0.43).

## Discussion

This review found no overall benefit when placing perinephric drains prophylactically during renal transplantation, including when assessing need for re-intervention post-operatively. Similarly, this review found no overall benefit of prophylactic drainage on reducing superficial or deep wound complications. Finally, there is not good evidence that perinephric drain placement is associated with improved graft survival outcomes at 12 months post renal transplantation.

Current literature demonstrates the range of complications associated with prophylactic drain insertion. One prospective study suggests that surgical site infection risk is increased when drains are inserted during general surgery procedures (OR 2.41, 95% CI 1.32–4.30, *p* = 0.004), however less of an effect is seen in vascular and orthopaedic surgery [4]. Furthermore, a systematic review and meta-analysis of twelve randomised controlled trials involving 1763 patients showed patients who underwent drainage had significantly higher pain scores as measured by the visual analogue scale (MD 10.08, 95% CI 5.24 to 14.92; *p* < 0.00001) [5]. There are limited studies reporting the incidence of bleeding and iatrogenic visceral injury secondary to perinephric drain placement. In one case series of deep pelvic collection drainage, a 2% haemorrhage rate was reported [16]. Fluid collections within the liver parenchyma may be amenable to percutaneous drainage, however this carries a reported 4% risk of major complications such as hepatocolic fistula creation, biliary peritonitis, and arterioportal fistula formation [17–19]. For retroperitoneal perinephric drains, a treatment failure rate exceeding 30% has been reported, often due to drain malposition [17].

Early post-operative collections such as seromas and haematomas occur post-transplant but the majority are discovered incidentally and are usually managed conservatively. The incidence of post operative surgical site haemorrhage detected by imaging and associated with a concurrent serum haemoglobin drop of more than 20 g/L over a 24 h period is relatively low (4.9%), with 90% of cases occurring within 1 day of implantation [20]. Collections more likely to require intervention such as urinomas, abscesses and lymphoceles, typically present later in the post-operative course, and the association with drain insertion is unclear. Lymphoceles in particular are common post renal transplant, with an incidence of 0.6%–51% reported in the literature, and 6.4% according to one recent retrospective study [21]. Urine leak has a reported incidence of 0.6%–6% and generally appears in the early post-transplant period [22, 23].

There have been two similar reviews in this area published previously. In 2019, D’Souza et al. showed that drain placement is associated with a higher incidence of peri-transplant fluid collections (RR 0.62; 95% confidence interval, 0.42–0.90), however no significant difference in the development of wound related complications [24]. A later review by Zawistowski et al. provided an update with the inclusion of a 2021 retrospective single-centre cohort study by Farag et al. The primary end-point in the Zawistowski meta-analysis was also perigraft collections [1]. No significant difference was seen between drain-free and drainage groups (pooled unadjusted OR = 0.77, 95% CI: 0.28–2.17). Similarly, there was no statistically significant difference in the secondary end points of surgical site infection, lymphocele, haematoma, and wound dehiscence between patients who did or did not receive prophylactic drainage. This review provides the most recent and extensive review of the current literature assessing the role of prophylactic perinephric drainage on short and long term clinically significant complications post kidney transplant. While previous reviews focused on the incidence of common post-operative complications, these are not necessarily clinically significant, as not all collections require drainage. By focusing our primary outcome on reintervention rate for post-operative collections, we aimed to better demonstrate the clinical significance of prophylactic drainage on renal transplant patients. More generally, the search criteria were robust and consistent across a range of generic and transplant-specific databases, with no language or time-period restrictions applied during article selection. All studies were rated as “good quality” when rated for quality via the Newcastle-Ottawa Score.

However, this review and analysis has several limitations. Given the retrospective nature of the studies identified in the literature, it is not possible to confidently demonstrate causality between our exposure (drain placement) and outcome (reintervention rate) of interest. The control groups included in the studies (no drain placement) would likely also be affected by selection bias. For instance, the Derweesh et al. study shows significant differences between the groups with respect to patient body mass index (BMI) and immunosuppression use (specifically sirolimus). These are both factors which are known to affect wound healing, surgical site infection, and wound complications specifically in renal transplantation, therefore the results cannot reliably be interpreted due to the selection bias present in the cohorts [25, 26]. Owing to the small number of studies included in this analysis (less than 10), publication bias could not be accurately assessed using Egger’s regression test for funnel plot asymmetry [27]. We found significant heterogeneity in the reporting of outcomes, and so meta-analyses were performed where specific outcomes were published. We also intended to record outcomes such as post-operative pain around the wound or drain site, opiate usage, length of hospital stay, and overall mortality, however these data were not available in the published literature in relation to drain use. Analysis of these outcomes would allow us to more effectively examine the complications associated with drain insertion, however due to the lack of availability we were not able to do so. Patient-reported outcomes and measures following drain insertion in particular would be an important aspect of drain insertion to assess and report upon, and one which we advocate should be investigated in future prospective studies. Regarding Figure 3, we intended to include Cimen et al. results in our meta-analysis, however were unable to contact the authors to obtain raw data to include in the meta-analysis. This represents a drawback to our review because Cimen et al. found lower odds of peri-graft collection, thus favouring drain insertion (*p* = 0.01). Finally, there was heterogeneity in the definitions of parameters such as “wound complications,” whereby authors divided into either clinically significant vs. not significant, or superficial vs. deep, or specifically looking at individual complications such as surgical site infection, wound dehiscence, or superficial wound collection. We therefore only included data from studies where we were confident that the data reflected the specific outcomes of interest described above.

One of the key rationales for intraoperative drain placement is pre-emptive control of post-operative collections such as lymphocele, seroma, haematoma, urinoma or infected tissue fluid. Ongoing monitoring for bleeding and infective collection around the graft site are the main indication for routine placement of a perinephric drain, however placement of the drain itself is associated with risks. In a meta-analysis of 28 randomised trials involving 3,659 patients, Gurusamy et al. showed that a drain-free approach to open cholecystectomy was associated with significantly lower wound infection rates, and no difference in the incidence of post-operative abdominal collection [28]. Partly as a result of this, drains are now no longer placed for uncomplicated open cholecystectomy operations. Furthermore, a single-centre experience of combined liver-kidney transplants showed no difference in the incidence of superficial/deep wound complications, collection size, intervention rate, graft failure, and overall patient survival between drainage and non-drain patient cohorts [29].

Better access to cross-sectional imaging provides a non-invasive tool for surgeons to utilise in the investigation for post-operative collections. Ultrasound provides accurate assessment of vascular flow to the graft, and can assess the presence of perinephric fluid collection and associated graft parenchymal compression [30]. Imaging is not always performed routinely, however in association with symptoms such as fever or pain, signs of graft failure such as high serum creatinine, ipsilateral leg swelling or hydronephrosis, drainage of these collections is indicated [31].

Current practice also shows a variety approaches to prophylactic drainage. In 2020, a survey of 43 renal transplant surgeons across Australia and New Zealand revealed 61% of surgeons practising routine drain insertion, while 21% rarely inserted drains [32]. A more recent (2023) survey of UK-based transplant surgeon practices suggests over two-thirds of respondents routinely insert one drain, while 8.3% indicated insertion of two or more drains on a routine, prophylactic basis. Only one-fifth of surgeons insert drains selectively as reported in this study [33]. This suggests the need for a paradigm shift in how prophylactic drainage in renal transplantation is viewed, especially in the absence of overwhelming evidence supporting its impact on favourable post-operative outcomes.

Given the lack of clear benefit of placing perinephric drains intraoperatively during renal transplantation, negative impact on patient experience, and the potential risks, we advocate for a an approach whereby drains are only placed for specific indications on a case by case basis. Prospective data is needed to support this position, and trial-level evidence is warranted to support or discourage routine perinephric drainage.

## Data Availability

The raw data supporting the conclusions of this article will be made available by the authors, without undue reservation.

## APPENDIX 1

Transplant Library Search URL: https://ovidsp.ovid.com/ovidweb.cgi?T=JS&NEWS=N&PAGE=main&SHAREDSEARCHID=3TvHv11Dt4cw1NlxqWFB6MvMmZDjDjslUOaG2iETazua2DxHJlBL1wKyGfIVYFHQn.

Medline Search URL: https://ovidsp.ovid.com/ovidweb.cgi?T=JS&NEWS=N&PAGE=main&SHAREDSEARCHID=31LygHgPjlVo7WTjuxhYBSXyljJHgkIVLLuds7cIY2hvyaKpJoHIjjvfZJJeXOJf5.

Embase search URL: https://ovidsp.ovid.com/ovidweb.cgi?T=JS&NEWS=N&PAGE=main&SHAREDSEARCHID=6sWADoa0ntU0FTQmkdaeO5QBXlCWubLBzIi7HG9w9P3AEztaoVShf2nSuZ4TDUKCJ.

Cochrane search URL: https://www.cochranelibrary.com/advanced-search/search-manager?p_p_id=58_INSTANCE_MODAL&p_p_lifecycle=0&p_p_state=normal&saveLastPath=false&_58_INSTANCE_MODAL_redirect=%2Fadvanced-search%2Fsearch-manager.
